# Aberrant aggressive behavior in a mouse model of Angelman syndrome

**DOI:** 10.1038/s41598-020-79984-7

**Published:** 2021-01-08

**Authors:** Lilach Simchi, Hanoch Kaphzan

**Affiliations:** Laboratory for Neurobiology of Psychiatric Disorders, Sagol Department of Neurobiology, University of Haifa, 199 Aba Khoushy Ave., Mt. Carmel, 3498838 Haifa, Israel

**Keywords:** Autism spectrum disorders, Social behaviour, Autism spectrum disorders

## Abstract

Angelman syndrome (AS) is a genetic neurodevelopmental disorder due to the absence of the E3-ligase protein, UBE3A. Inappropriate social interactions, usually hyper-sociability, is a part of that syndrome. In addition, clinical surveys and case reports describe aggressive behavior in AS individuals as a severe difficulty for caretakers. A mouse model for AS recapitulates most of the human AS phenotypes. However, very few studies utilized this mouse model for investigating affiliative social behavior, and not even a single study examined aggressive behavior. Hence, the aim of the herein study was to examine affiliative and aggressive social behavior. For that, we utilized a battery of behavioral paradigms, and performed detailed analyses of these behaviors. AS mice exhibited a unique characteristic of reduced habituation towards a social stimulus in comparison to their wild-type (WT) littermates. However, overall there were no additional marked differences in affiliative social behavior. In contrast to the mild changes in affiliative behavior, there was a striking enhanced aggression in the AS mice compared to their WT littermates. The herein findings emphasize the use of AS mouse model in characterizing and measuring inappropriate aggressive behavior, and suggests these as tools for investigating therapeutic interventions aimed at attenuating aggressive behavior.

## Introduction

Angelman syndrome (AS) is a rare neurodevelopmental disorder with a prevalence of 1 in 15,000 live births^1^. AS is associated with severe developmental delay, susceptibility to epilepsy, absence of speech, motor abnormality, and a hallmark behavioral profile which includes frequent laughter, and an easily excitable personality^2,3^. The culprit behind AS is the loss of the maternally expressed ubiquitin-protein ligase E3A gene (*UBE3A*), mostly due to a deletion in the 15q11–q13 chromosomal region encompassing this gene^4,5^. *UBE3A* is imprinted in a tissue-specific manner, and specifically maternal expression disruption results in the absence of UBE3A in neurons^6,7^.

Bidirectional changes in UBE3A expression attracts considerable interest due to their linkage with autistic symptomatology^8–12^. However, in contrast to the usual phenotype of avoidance in autism, AS patients exhibit a unique hyper-social demeanor, which manifests in an excessive uninhibited desire to seek attention from both caregivers and strangers alike^13–16^. It was suggested that this inappropriate social seeking behavior accounts for some of the challenging behaviors in AS individuals, such as aggression^17–19^, which was commonly reported for AS affected individuals^17,20–22^. A review that examined AS behavioral phenotypes by reviewing 64 publications, mainly case reports, reported that 15% of them referred to aggressive behaviors^15^. Aggressive behaviors were estimated to occur three times more in AS than in individuals with a comparable level of intellectual disability^20^. Notably, this reported aggressive behavior is mostly manifested by mild level aggressive behaviors (e.g., hair pulling, grabbing) rather than full blown physical aggression^21^. Nonetheless, due to the burden on caretakers, it is necessary to study possible therapies that can diminish aggressive behavior. The ability to investigate the modification of aggressive behavior in AS is limited in humans, and require the use of valid animal models.

A major contribution to the comprehensive investigation of the pathophysiology of AS has been the development of AS mouse models. Previous studies showed that these mouse models recapitulate the various phenotypes found in humans with AS, such as motor dysfunction, susceptibility for seizures, and cognitive deficits^23–26^. While cognitive AS hippocampal-dependent deficits and motor performance have been extensively studied in AS mouse models, the striking social behavior characteristics associated with AS have been mostly neglected. To the best of our knowledge, only five studies thoroughly examined social behavior using an AS mouse model^27–31^. However, these studies focused primarily on the sociability aspect, and did not examine a broader range of social behaviors, such as aggression.

The herein study aimed to comprehensively characterize elements of both affiliative (sociability) and agonistic (offensive aggression) social behaviors in AS mouse model. Furthermore, we delineated additional essential social abilities, such as social recognition and social habituation.

## Results

### Sociability and social discrimination are unaltered in AS model mice

**Figure 1:**
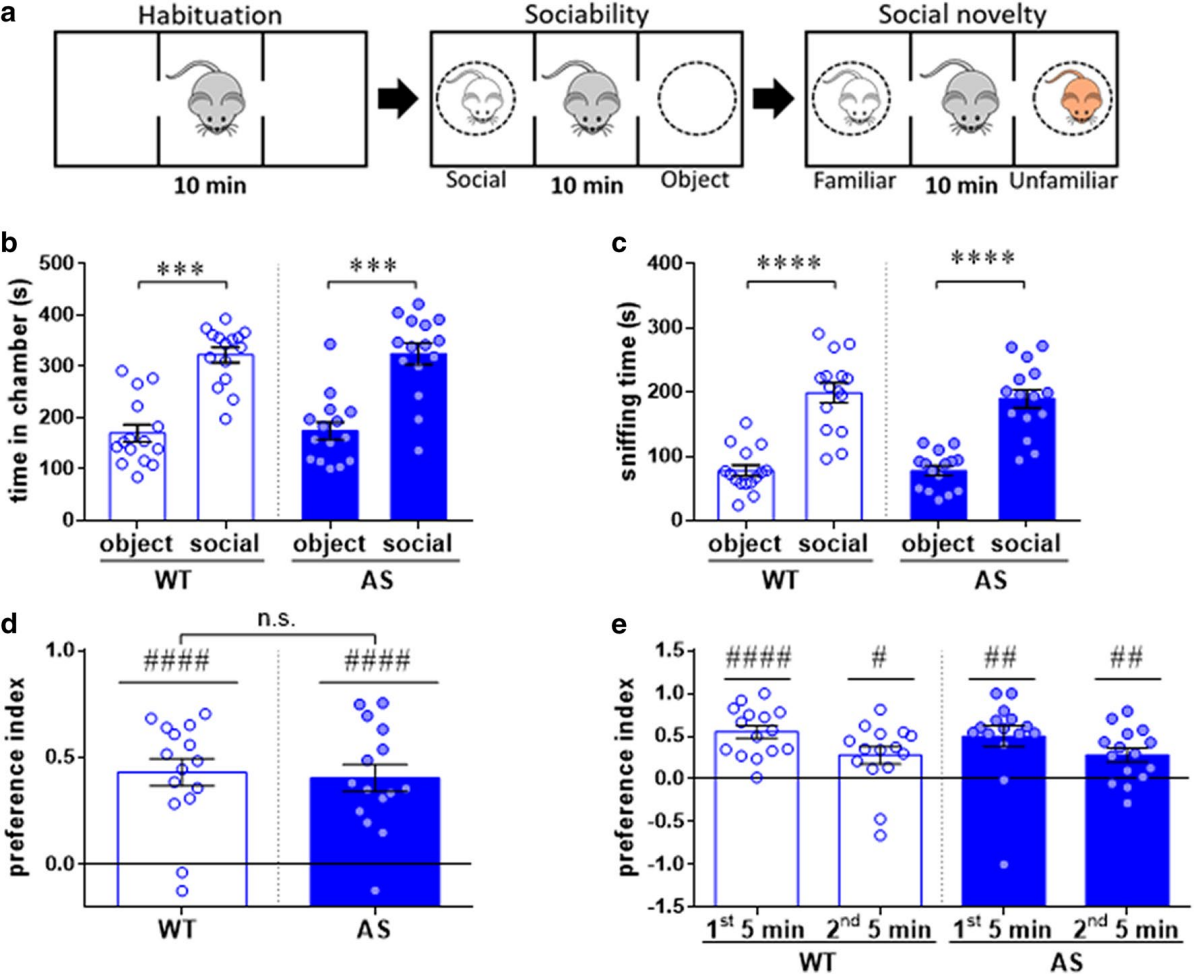
AS male mice do not display aberrant sociability in the three-chamber social test. (**a**) Illustration of sociability and social novelty tests. (**b**) Time spent in the chamber containing the novel mouse (‘social’) and the chamber containing the empty wire cage (‘object’). Both genotypes exhibit enhanced exploration of the social chamber. (**c**) Sniffing time of the novel mouse and the empty wire cage. Both WT and AS mice spend more time sniffing the social stimulus (novel mouse). (**d**) Social preference index (PI) towards the social stimulus. WT and AS mice exhibit a comparable social PI. (**e**) Social PIs are binned into 5-min bins. Both genotypes maintained positive social PI along the entire trial. Bar graphs represent mean ± SEM. N = 15 mice per each group (WT and AS). ***p < 0.001,****p < 0.0001, n.s. = non-significant; ^#^*p* < 0.05, ^##^*p* < 0.01, ^####^*p* < 0.0001 represent a difference from chance level.

To determine whether AS model mice recapitulate the hyper-sociability phenotype observed in AS-affected humans, we tested their preference to interact with a social stimulus (a novel mouse enclosed in a wire cage, ‘social’) over a non-social one (an empty wire cage, ‘object’), utilizing the three-chamber social test (Fig. 1a).

In order to eliminate confounding influences of side preference, we compared the time spent in each side chamber during the habituation trial, when both of the side chambers were empty [F_(1,28)_ = 0.14, p = 0.71; F_(1,28)_ = 0.21, *p* = 0.65 for genotype and side of chamber effects in 2way RM-ANOVA]. The results show no significant preexisting preference to either side in AS and their WT littermates. Additionally, the number of transitions between the central chamber into each of the side chambers did not exhibit any side preference [F_(1,28)_ 0.7, *p* = 0.41 for side of the chamber effect in 2way RM-ANOVA]. However, there was a trend for a genotype effect [F_(1,28)_ = 3.81, *p* = 0.06 for genotype effect in 2way RM-ANOVA] suggesting a difference in exploratory locomotor functioning between the genotypes^32^, or simply reflecting the motor deficits of AS mice as we and others previously showed^23,33^.

Behavioral analysis of the time spent in each of the side chambers (social vs. object) showed comparable social propensity between AS mice and their WT littermates. Both spent significantly more time in the chamber containing the social stimulus relative to the chamber containing the object [F_(1,28)_ = 43, *p* < 0.0001 for chamber type effect in 2way RM-ANOVA; Bonferroni's post-hoc comparisons: t_(28)_ = 4.68, *p* < 0.001; t_(28)_ = 4.6, *p* < 0.001 for WT and AS respectively] (Fig. 1b). Similarly, both were more engaged in sniffing the wire cage containing the stranger mouse than the dummy wire cage [F_(1,28)_ = 77.16, *p* < 0.0001 for sniffing-time effect in 2way RM-ANOVA; Bonferroni's post-hoc comparisons: t_(28)_ = 6.45, *p* < 0.0001; t_(28)_ = 5.97, *p* < 0.0001 for WT and AS respectively] (Fig. 1c). In order to avoid a possible artifact due to the motor deficits of AS mice, we next calculated the social preference index (PI)^34^. The social PI was significantly above the chance level (PI = 0) for both AS and WT, with no difference between the genotypes [t_(28)_ = 0.31, *p* = 0.76 in unpaired t-test; t_(14)_ = 6.74, *p* < 0.0001, t_(14)_ = 6.41, *p* < 0.0001 for WT and AS respectively in one sample t-test] (Fig. 1d). In addition, both genotypes showed a significant positive social PI along the entire test, also during the last 5 min [F_(1,28)_ = 0.06, *p* = 0.81 for genotype effect in 2way RM-ANOVA; for WT: t_(14)_ = 7.49, *p* < 0.0001, t_(14)_ = 2.76, *p* < 0.05, and for AS: t_(14)_ = 4.05, *p* < 0.01, t_(14)_ = 3.53, *p* < 0.01 for first 5-min and last 5-min respectively, in one sample t-test] (Fig. 1e). To conclude, these results indicate that AS mice exhibit normal levels of sociability in the three-chamber social test, which coincides with previous studies^28,29^.

**Figure 2:**
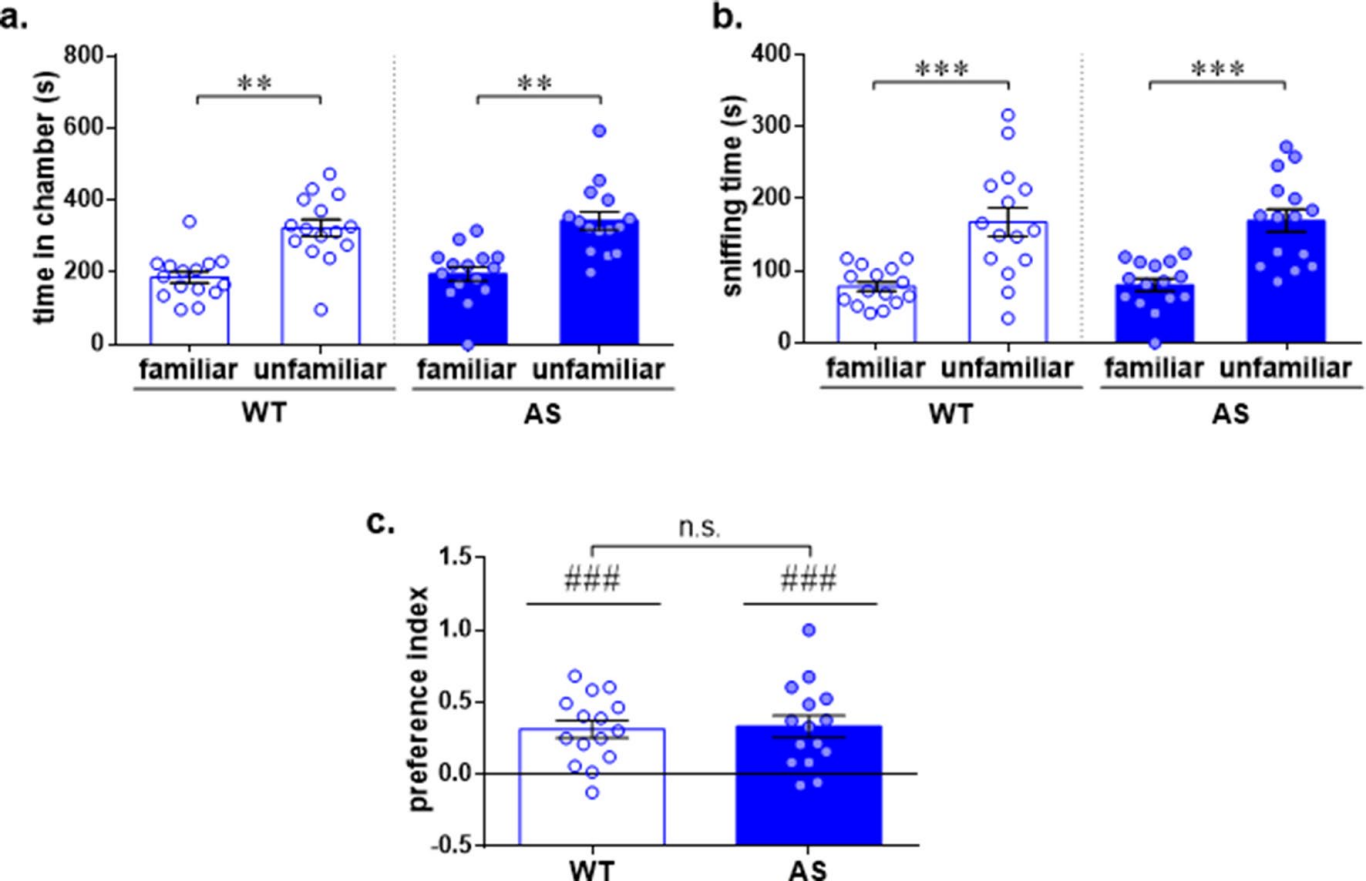
AS male mice display intact social discrimination in social novelty three-chamber social test. (**a**) Time spent in the chamber containing the familiar mouse and the chamber containing the unfamiliar mouse. WT and AS mice spend more time in the chamber containing the unfamiliar stimulus. (**b**) Time spent sniffing each stimulus mouse. WT and AS demonstrated comparable enhanced sniffing of the unfamiliar stimulus mouse relative to the familiar mouse. (**c**) Social novelty preference index (PI). AS mice display a comparable positive social novelty PI toward the unfamiliar mouse. Bar graphs represent mean ± SEM. N = 15 per each group (WT and AS). **p < 0.01, ***p < 0.001, n.s. = non-significant; ^###^*p* < 0.001 represents a difference from chance level.

Next, we performed the social novelty test. This test examines the ability of AS mice to discriminate between familiar and novel social stimuli using the sequential session of the three-chamber social test. Following the sociability test, a novel stimulus mouse (‘unfamiliar’) was presented in the previously empty wire cage (Fig. 1a). In the social novelty test, WT and AS mice showed similarly longer exploration of the unfamiliar-associated chamber compared to the familiar-associated chamber [F_(1,28)_ = 24.36, *p* < 0.0001 and F_(1,28)_ = 2.63, *p* = 0.12 for chamber type and genotype effects respectively, in 2way RM-ANOVA; Bonferroni's post-hoc comparisons: t_(28)_ = 3.35, *p* < 0.01; t_(28)_ = 3.63, *p* < 0.01 for WT and AS respectively] (Fig. 2a). Similarly, both genotypes exhibited longer sniffing duration of the wire cage containing the unfamiliar mice [F_(1,28)_ = 37.42, *p* < 0.0001 for sniffing time effect in 2way RM-ANOVA; Bonferroni's post-hoc test comparisons: t_(28)_ = 4.33, *p* < 0.001; t_(28)_ = 4.32, *p* < 0.001 for WT and AS respectively] (Fig. 2b), and the positive social novelty PI was comparable between AS and WT mice [t_(28)_ = 0.19, *p* = 0.85 in unpaired t-test between genotypes; t _(14)_ = 5.16, *p* < 0.001, t _(14)_ = 4.36, *p* < 0.001 for WT and AS respectively in one sample t-test] (Fig. 2c). These results suggest that AS mice clearly distinguish between two conspecifics based on familiarity discrimination, similar to their WT littermates.

### AS male mice display impaired social habituation response

**Figure 3:**
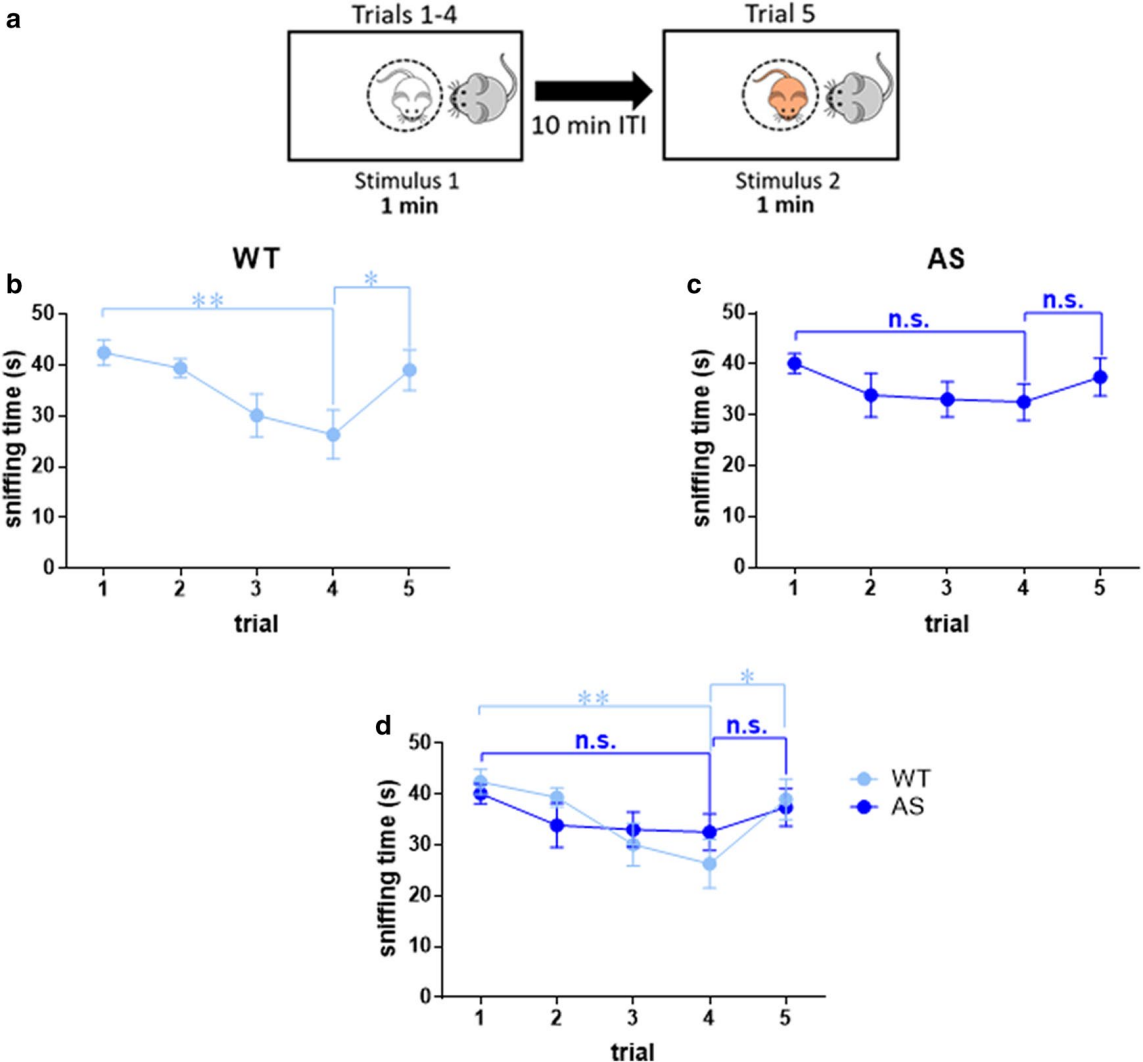
AS male mice exhibit impaired social habituation in social habituation\dishabituation test. (**a**) Illustration of social habituation/dishabituation test. (**b**–**d**) Social exploration time in each of the five trials. (**b**) Sniffing time of stimulus mouse by WT mouse. WT mice show habituation to the repeatedly introduced stimulus mouse, and dishabituation towards the novel stimulus mouse. (**c**) Sniffing time of stimulus mouse by AS mouse. AS mice do not show habituation to the repeatedly introduced stimulus mouse, nor dishabituation to the novel stimulus mouse. (**d**) Concatenated graph of graphs **a** and **b**. Line graphs represent mean ± SEM. N = 12 mice per each group (WT and AS). *p < 0.05, **p < 0.01 and n.s. = non-significant represent differences between trials within groups.

Aiming to substantiate our investigation regarding the aforementioned findings, we further conducted a social habituation/dishabituation assay. Unlike the social novelty test, in which stimuli are presented simultaneously, this assay incorporates a delay period between consecutive stimulus presentations. Thus, this assay defines recognition based on habituation and dishabituation responses rather than novelty preference (Fig. 3a). WT mice demonstrated a habituation response to the four repeated sequential introductions of the same stimulus mouse (‘habituation phase’). This manifested by a significant decline in the social exploration between the first and fourth trials [F_(4,88)_ = 4.94, *p* < 0.01 for trial effect in 2way RM-ANOVA; Bonferroni's post-hoc test for comparison between trial 1 and trial 4, t_(88)_ = 3.69, *p* < 0.01]. However, following the fifth encounter with a novel stimulus mouse (‘dishabituation phase’), WT mice demonstrated a complete reemergence of vigorous investigation towards that novel stimulus [Bonferroni's post-hoc test for comparison between trial 4 and trial 5, t_(88)_ = 2.91, *p* < 0.05] (Fig. 3b). This increase confirms that the observed reduction along the first four encounters is derived from habituation due to familiarization rather than a general reduction in exploration over time.

In contrast, AS males did not display marked habituation to the re-exposed social stimulus [Bonferroni's post-hoc test for comparison between trial 1 and trial 4: t_(88)_ = 1.73, *p* = 0.87]. This probably precluded the typical augmentation in social investigation during the fifth encounter with the novel mouse [Bonferroni's post-hoc test for comparison between trial 4 and trial 5, t_(88)_ = 1.12, *p* > 0.99] (Fig. 3c,d). Despite the above findings, there were no interaction effects between genotype and social exploration time [F_(3,66)_ = 1.5, *p* = 0.22; F_(1,22)_ = 2.3, *p* = 0.15 for habituation and dishabituation, respectively, in 2way RM-ANOVA]. Nonetheless, the data suggest an overall impairment in habituation to social stimuli in AS model mice.

### AS model mice display a minor alteration in long-term social discrimination

**Figure 4:**
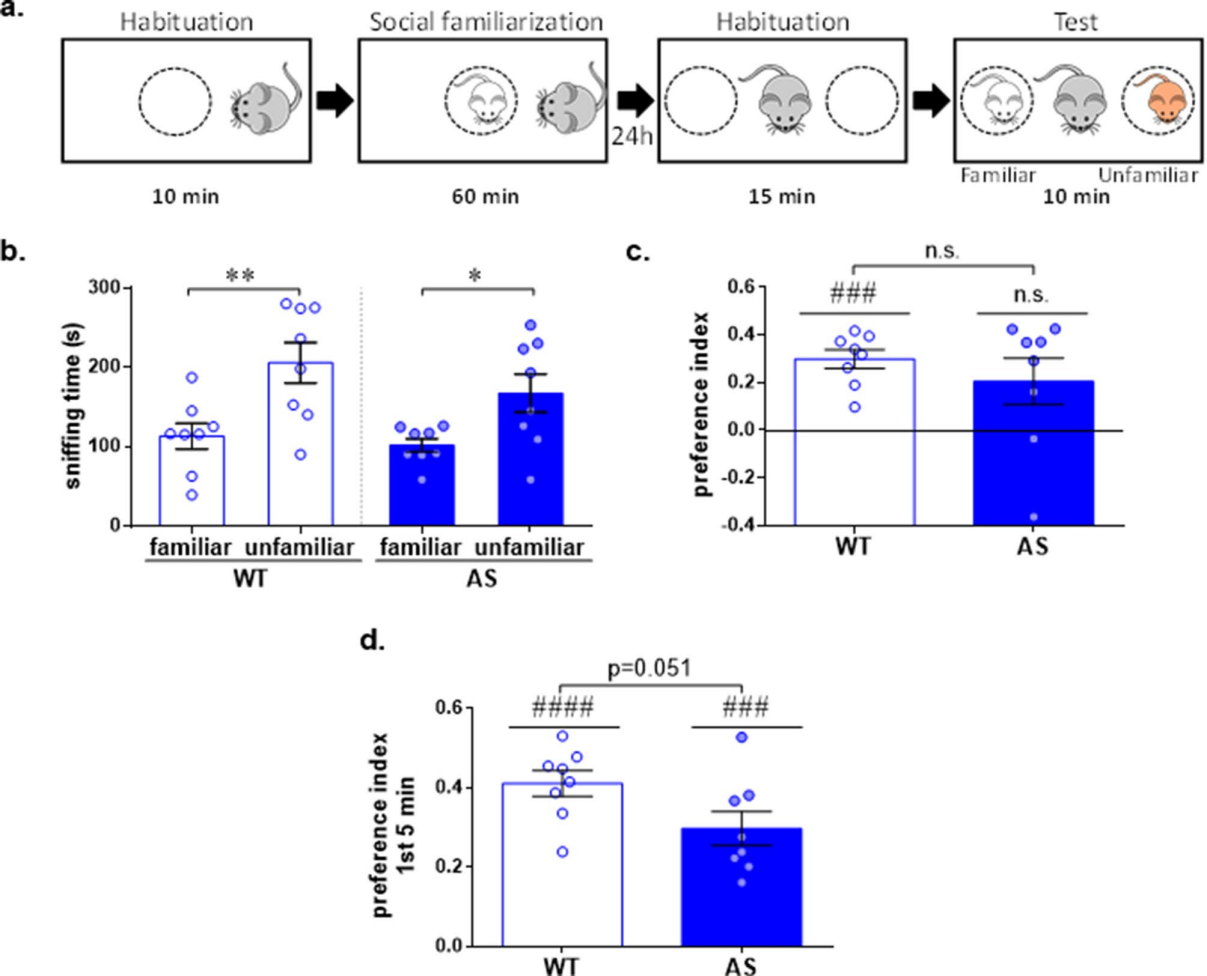
AS model mice display a minor alteration in long-term social discrimination memory (SDM). (**a**) Illustration of long-term SDM test. (**b**) Time spent sniffing the familiar mouse and the unfamiliar mouse, 24 h after the exposure to the familiar mouse. WT and AS mice similarly spend more time sniffing the unfamiliar mouse than the familiar mouse. (**c**) Long-term SDM preference index (PI) towards the unfamiliar mouse. WT mice display positive long-term SDM PI, while AS mice do not. However, the long-term SDM PI does not differ between genotypes. (**d**) Long-term SDM PI during the first 5 min of the test. Both genotypes showed a positive PI during the first 5 min, yet WT mice exhibit a trend for a higher PI compared to AS littermates. Bar graphs represent mean ± SEM. N = 8 mice per each group (WT and AS). *p < 0.05, **p < 0.01; ^###^*p* < 0.001, ^####^*p* < 0.0001, n.s. = non-significant, represent differences from the chance level; ITI = inter-trial interval.

Previous studies in AS model mice have reported profound deficits in some of the long-term memory paradigms^23–25,35^. However, long-term social memory has never been studied in AS mice. Owing to the fact that AS mice demonstrate typical social discrimination ability in social novelty test, we modified this paradigm in order to assess their long-term social discrimination memory (SDM). For that, we introduced a pre-exposed stimulus mouse (24 h post-exposure) in parallel to a novel stimulus mouse (Fig. 4a). Both WT and AS mice exhibited a more extensive investigation of the unfamiliar stimulus compared to the familiar one, with no difference between the genotypes [F_(1,14)_ = 1.12, *p* = 0.31 and F_(1,14)_ = 28.65, *p* < 0.001 for genotype and sniffing time effects respectively, in 2way RM ANOVA; Bonferroni's post-hoc test comparisons: t_(14)_ = 4.43, *p* < 0.01, t_(14)_ = 3.14, *p* < 0.05 for WT and AS respectively] (Fig. 4b). This was reflected in a comparable PI for long-term SDM between WT and AS mice [t_(14)_ = 0.89, *p* = 0.39 in unpaired t-test] (Fig. 4c), although only the WT mice displayed long-term SDM PI that was significantly higher than the chance level [t_(7)_ = 7.71, *p* < 0.001, t_(7)_ = 2.1, *p* = 0.07 for WT and AS respectively in one sample t-test]. Notably, the long-term SDM PI during the first 5 min was significantly different from the chance level in both genotypes [t_(7)_ = 12.79, *p* < 0.0001, t_(7)_ = 6.97, *p* < 0.001 for WT and AS respectively in one sample t-test], but the PI of the WT showed a strong trend for being higher than that of the AS [t_(14)_ = 2.13, *p* = 0.051 in unpaired t-test] (Fig. 4d). Taken together, these findings suggest that AS mice demonstrate a long-term social discrimination ability along the first 5 min of the test, yet not comparable to the level of their WT littermates.

### AS model mice show increased offensive behavior

**Figure 5:**
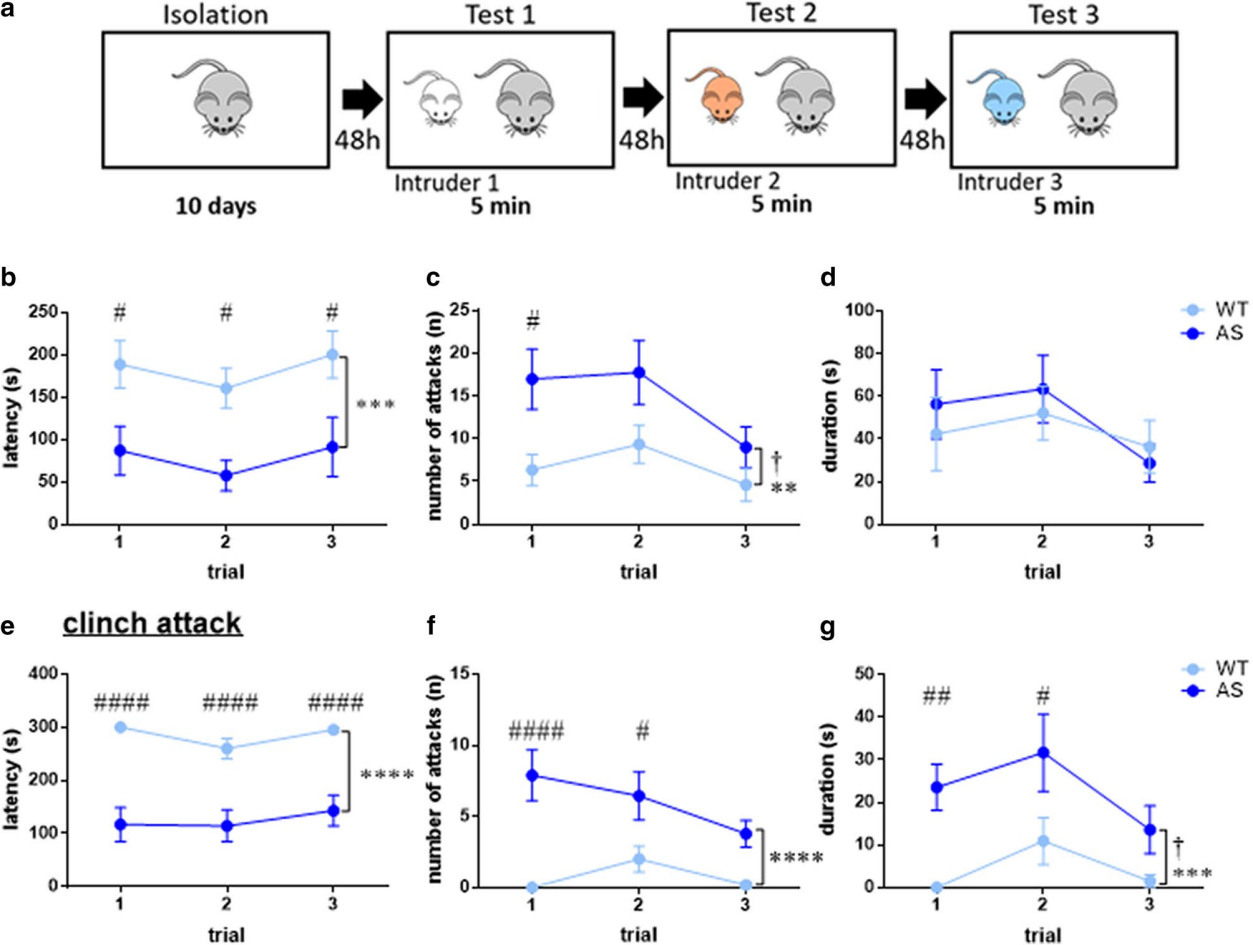
AS male mice display enhanced aggression in the resident-intruder test. (**a**) Illustration of the resident-intruder test. (**b**–**d**) Overall aggressive behavior is enhanced in AS mice. (**b**) The latency to the first attack. (**c**) Number of attacks during 5 min. (**d**) Total accumulative duration of attacks. (**e**–**f**) excessive uncontrolled aggression (clinch) is markedly predominant in AS male mice. (**e**) Latency to the first clinch attack. (**f**) Number of clinch attacks during 5 min. (**g**) Cumulative duration of clinch attacks. Line graphs represent mean ± SEM. N = 11 mice, N = 9 mice for WT and AS, respectively. ^#^*p* < 0.05, ^##^*p* < 0.01, ^####^*p* < 0.0001 represent a genotype difference for each trial. **p < 0.01, ***p < 0.001, ****p < 0.0001 represent an accumulative genotype effect. ^†^*p* < 0.05 represents a  trial effect.

In order to study inter-male offensive aggression, we adapted the resident-intruder test. In this assay, a group-housed male mouse (‘intruder’) was introduced into the home cage of an isolated mouse (‘resident’) for 5 min. To address experience-dependent alterations, we repeated this experiment three times with a 48 h inter-trial interval (Fig. 5a). In almost all of these experiments, aggression was initiated by the resident. Only in two cases, severe aggression was initiated by the intruder, and these two resident mice were excluded from the data. Remarkably, these two resident mice were AS, and there was not a single case in which the intruder initiated an attack on a WT resident mouse.

The resident-intruder experiments demonstrated a remarkably enhanced offensive behavior of AS mice compared to their WT littermates. The latency for an attack was much shorter for AS mice than for their WT littermates [F_(1,18)_ = 15.57, p < 0.001 for genotype effect in 2way RM-ANOVA]. This shorter latency for the first attack in AS was found in each of the three exposure trials [Bonferroni's post-hoc comparison between genotypes: t_(54)_ = 2.63, *p* < 0.05; t_(54)_ = 2.66, *p* < 0.05; t_(54)_ = 2.81, *p* < 0.05 for the first, second and third exposures respectively] (Fig. 5b). In addition, AS mice exhibited an overall increased number of attacks [F_(1,18)_ = 9.64, *p* < 0.01 for genotype effect, in 2way RM ANOVA], but this difference between the genotypes dissipated along the trials [F_(2,36)_ = 4.53, *p* < 0.05 for trial sequence effect, in 2way RM ANOVA; Bonferroni's post-hoc between genotypes: t_(54)_ = 2.9, *p* < 0.05, t_(54)_ = 2.29, *p* = 0.08, t_(54)_ = 1.19, *p* = 0.72 for the first, second and third exposures, respectively] (Fig. 5c). Interestingly, in spite of the overall increased aggression exhibited by AS, the attack duration once it was already launched was comparable to the duration in the WT mice [F_(1,18)_ = 0.16, *p* = 0.7 for genotype effect, in 2way RM ANOVA] (Fig. 5d). A different quality of attack is the clinch attack, which is considered a severe form of uncontrolled aggression^36–38^. Clinch attack is defined as a rapid, salient attack, which consists violent kicking with the hind-paws and relative long-lasting bites, directed preferentially toward the intruder’s back, neck and flanks. During this attack, the resident mice grasp the frontal-dorsal part of the intruder, rolling over and wrestle while trying to save his own back safe^37,39^. Segregating the clinch attacks revealed remarkable enhanced aggression in AS in all parameters (Fig. 5e–g). AS mice consistently initiated the first attack on the intruder faster than their WT littermates throughout the consecutive exposure trials from the first to the last [F_(1,18)_ = 76.24, *p* < 0.0001 for genotype effect in 2way RM-ANOVA; Bonferroni's post-hoc comparisons: t_(54)_ = 6.13, *p* < 0.0001; t_(54)_ = 4.87, *p* < 0.0001; t_(54)_ = 5.09, *p* < 0.0001, for first, second and third exposures, respectively] (Fig. 5e). However, the parameters of frequency and duration appeared to be affected by the previous encounters, and showed slight diminishment with exposures (Fig. 5f,g). The frequency of clinch attacks showed a trend for diminishing along the exposure trials [F_(1,18)_ = 33.72, *p* < 0.0001; F_(2,36)_ = 2.9, *p* = 0.07 for genotype and trial sequence effects respectively, in 2way RM-ANOVA]. Thus, the difference between genotypes faded along the exposures [Bonferroni's post-hoc comparisons: t_(54)_ = 5.31, *p* < 0.0001; t_(54)_ = 2.99, *p* < 0.05; t_(54)_ = 2.42, *p* = 0.057 for first, second and third exposures, respectively] (Fig. 5f). Similarly, the duration of the clinch attacks declined along trials, and the difference between the genotypes gradually disappeared [F_(1,18)_ = 18.42, *p* < 0.001; F_(2,36)_ = 4.04, *p* < 0.05 for genotype and trial sequence effects respectively, in 2way RM-ANOVA; Bonferroni's post-hoc comparisons: t_(54)_ = 3.26, *p* < 0.01; t_(54)_ = 2.88, *p* < 0.05; t_(54)_ = 1.68, *p* = 0.3 for first, second and third exposures, respectively] (Fig. 5g).

Interestingly, we also examined the social exploration element of sniffing within the aggression test of the resident-intruder paradigm. This affiliative behavior of sniffing, which contrasts the aggressive behavior, coincided with the aggression results. Overall, AS mice were considerably less engaged in social sniffing, and showed an increased latency for sniffing activity, together with a decreased number of sniffing events and decreased time spent in sniffing activity [F_(1,18)_ = 8.74, *p* < 0.01; F_(1,18)_ = 8.93, *p* < 0.01; F_(1,18)_ = 9.85, *p* < 0.01 genotype effects for latency, frequency and duration respectively in 2way RM-ANOVA] (Supplementary Fig. S1).

It is critical to note that the weight of the AS resident mice was higher compared to the WT littermates [t_(18)_ = 4.45, *p* < 0.001 in unpaired t-test]. However, no correlation was found between the weights of the resident mice to the aggression levels within each genotype [e.g. Pearson correlation between weight and the frequency of the clinch attacks during the second trial: r = -0.43, *p* = 0.18; r =  − 0.13, *p* = 0.74 for WT and AS respectively] (Supplementary Table S1). Also, analysis of covariance (ANCOVA) corrected to the weight, did not annul the significant differences in most of the aggression parameters between the genotypes [e.g. F_(2)_ = 3.7, *p* < 0.05 for the frequency of the clinch attacks during the second trial in ANCOVA]. Altogether, these data demonstrate that AS mice display a marked accelerated and enhanced offensive aggressive behavior.

## Discussion

One of the cardinal features of autism spectrum disorders (ASD) are the persistent difficulties in social communication and interaction with others^40^. It has been suggested that the *UBE3A* gene dosage may have a causative effect upon the expression of autistic traits^8–11,41^. In fact, inherited maternal duplications and triplications of the sequence encompassing *UBE3A* gene (15q11–13), where UBE3A is overexpressed, are amongst the most common genome copy number variations observed in ASD^42^. On the other hand, individuals with AS, a condition where UBE3A is almost not expressed in the brain, also display autistic characteristics^13,43, 44^. Nevertheless, despite the display of some typical autistic features in AS individuals, such as the lack of speech and inappropriate social behavior, they exhibit a unique inappropriate hyper-social disposition, opposite to the one usually found in ASD^13,14^. While few studies examined the affiliative social behavior in AS mouse model, as far as we know, no study investigated the agonistic social aspects in this model. Hence, we sought to investigate whether social behavior in general, and social agonistic behavior in particular, are affected in AS model mice. In accordance with previous studies, our findings show that AS model mice have a social preference comparable to their WT littermates in the three-chamber social test, during a single 10 min trial (Fig. 1a–d)^28,29^. These results do not concur with the hyper-sociability phenotype found in AS patients^14^. It was suggested that the isolated genetic modification by which the mouse model was generated, does not reflect the usual larger deletions in humans, and thus does not recapitulate the hyper-sociability phenotype^28^. Besides the difference between the genetic mechanisms in AS humans to those in model mice, it is possible that the three-chamber social assay is a useful paradigm for evaluating gross social preference deficits, but not so sensitive for milder quantitative differences in sociability levels^34^. In addition, the congruence between mice models studies is also far from being perfect. In contrast to the aforementioned results, two previous studies demonstrated profound alteration in sociability, utilizing the same animal model and paradigm as the current study. Interestingly, the findings of these studies stand in stark contrast to each other^27,30^. On the one hand, AS mice on a 129 background exhibit aberrant decreased level of sociability compared to their WT counterparts, in the three-chamber social test^30^. Conversely, the second study showed that AS mice on a FVB/NJ background display a more prolonged preference compared to their respective WT littermates, when the 10 min trial is divided into two successive 5 min time-bins^27^. However, in our hands neither of these findings was reproduced (Fig. 1b-d,e, respectively). One possible explanation for this disparity is the different genetic background utilized in each study (129^30^, FVB/NJ^27^, C57BL/6 in the herein study)^27^. This is supported by a previous study that exhibited differences in sociability levels in this task between these three stains (FVB/NJ < C57BL/6 < 129)^32^. Age is an additional important confounding variable concerning examining AS mice sociability^31^. Nonetheless, the two aforementioned studies and the herein study utilized mice at approximately the same age group (8–13 weeks)^27,30^. All of the above emphasize the need for further studies aimed at examining the consistency of social preference along time with age and strain-specific considerations.

The ability to recognize and discriminate between conspecific individuals forms the foundation for appropriate social behavior across social species^45^. Interestingly, although AS mice were observed to possess a behavioral response indicative of normal social recognition in social novelty test (Fig. 2), surprising differences between AS and WT mice were detected during the social habituation/dishabituation test (Fig. 3). AS mice failed to demonstrate the expected repressive effects of repeated exposures to the same conspecific, known as habituation. One parsimonious explanation regarding this discrepancy may be ascribed to methodological differences between the two paradigms. Contrary to the social novelty test, in which both stimuli are presented simultaneously, in the social habituation/dishabituation test the familiar and unfamiliar stimuli are introduced sequentially with 10 min intervals. These differences were suggested as an explanation for making the social habituation/dishabituation test a harder task in comparison to the social novelty test^45^. However, since AS mice demonstrated overall intact social recognition ability following 24 h interval in long-term SDM test (Fig. 4d), the lack of decay in social exploration in the social habituation/dishabituation test probably indicates impaired social habituation, rather than social recognition deficit per se. This finding contributes to a more nuanced view of the recently emerging evidence regarding the hyper-social demeanor in AS model mice^27^, which is also manifested in human AS individuals.

The social interactions that are critical for the survival of the individual in society are composed not only of affiliative social behaviors, but also of appropriate agonistic behavior and aggression^46^. Our results show that offensive aggression towards a conspecific is higher in AS mice compared to their WT littermates in almost all parameters (Fig. 5). Furthermore, a closer inspection of the aggressive behavior at the interactions with an intruder revealed not merely quantitative but also qualitative differences. Relative to WT mice, the attacks committed by AS mice were clinch attacks that are characterized by being more severe and injurious (Fig. 5e–g)^36–38^. Offensive aggression behaviors, including clinch attacks, are intended to inflict harm and can potentially have destructive consequences not only for the victim, but for the aggressor as well^47^. Thus, inhibitory control mechanisms have been developed to tightly regulate aggressive behavior for the sake of minimizing their adverse outcomes. Processing of external social cues aims to assure the appropriate behavioral response in complex social situations. This includes the attenuation of aggressive behavior to more moderate and reasonable levels^37,48^. Our results suggest that AS mice exhibit an excessive and uncontrolled aggressive behavior, which is in agreement with previous descriptions of human AS patients in the literature^17,20–22^. However, a major challenge when interpreting this striking result is to identify the possible causes underlying the observed acceleration of the offensive aggression. Since aggression comprises numerous facets, a complex of variables may be implicated in this behavior, including the integration of social clues, motivation, sensory perception, anxiety, etc^49–51^.

The modular effect of these factors is regulated by a structurally and functionally highly interconnected neuronal network that controls aggressive behaviors. This network includes the periaqueductal gray, lateral septum, amygdala, the prefrontal cortex (PFC), and the ventromedial nucleus of the hypothalamus^48^. Some of these regions were found to be affected in AS. For example, the excitability of PFC was found to be differentially altered in distinct subregions. In layer-V pyramidal neurons of the infralimbic PFC there was an enhancement of excitability^52^, while the layer-V pyramidal neurons of the medial PFC excitability was reduced^53^. In the amygdala, also a brain region related to aggression, it was found that the number of parvalbumin-positive interneurons was reduced in AS mice^54^. In addition to the neuroanatomy of aggression, a large body of animal neurobehavioral based research provided a detailed view on the neuromolecular mechanisms involved in the above regulatory neurocircuitry, such as monoamines (serotonin and dopamine), and steroid hormones (corticosterone and estrogen)^47^. These molecular pathways are also affected in Angelman syndrome, and have the potential to affect the aggression-related neurocircuitry. Notably, a previous study has shown that the regulation of several neurotransmitters, such as serotonin (5-HT) and dopamine (DA), have been differentially dysregulated in various brain regions of AS mice^55^. Moreover, AS mice also exhibit downregulation of the glucocorticoid receptor (GR) signaling pathway, results in increased susceptibility to stress and anxiety^56^. A subsequent study demonstrated that treatment with fluoxetine, a selective serotonin reuptake inhibitor (SSRI), partially restored the impaired glucocorticoid signaling and rescued anxiety-like behavior in AS mice^54^. These key-signaling molecules are implicated as possible contributors for AS maladaptive aggression. This warrants further exploration to provide a more comprehensive characterization of AS inappropriate agonistic behavior, as well as the underlying neural and molecular mechanisms.

Interestingly, it seems as if the herein study exhibits two juxtaposed findings, the diminished habituation of pro-social behavior alongside enhanced aggression. However, It was previously suggested that increased pro-social behavior might actually accelerate the aggressive challenging behavior^18,19^. Furthermore, several studies claimed that such aggressive behaviors are often used as a communication method for protest, gaining or sustaining social interactions due to hyper-social disposition^18,57,58^. Further investigation of the interplay between the above phenotypic behaviors might shed light on the underlying neurobiological and genetic mechanisms in AS.

To conclude, we herein provide additional evidence for the hyper-social demeanor together with novel indications regarding excessive aggression in AS model mice that coincide with clinical reports. Aggressive behavior is one of the most challenging difficulties that caretakers are facing. Discovering therapies that alleviate aggressive behavior is desired, and this requires the use of animal models. Our study supports the use of AS mouse model for investigating therapeutic strategies aimed at attenuating aggression. Furthermore, aggression is a common symptom of multiple psychiatric and neurodevelopmental disorders, and studies in AS mice have the potential to extend their impact on these disorders as well.

## Methods

### Mice

AS model mice and their wild-type (WT) littermate controls were generated on a C57BL/6 background and genotyped using specific primers, as described previously^23^. Mice for experiments were bred from a female that was heterozygous for the deletion of *Ube3a* from a paternal origin (*Ube3a*^p−/m+^) and a WT male. Mice were housed in groups of 3–5 animals per cage. For affiliative social interaction experiments, mice were handled and habituated to the experimenter for a week prior to the start of the test. On the contrary, mice for aggression tests were singly housed for 10 days and were not handled or habituated. Four separate cohorts of AS mice and their WT littermates were used for each test; for three-chamber social test AS N = 15, WT N = 15; for habituation/dishabituation test AS N = 12, WT N = 12; for long-term social discrimination memory (SDM) test AS n = 8, WT n = 8; for resident-intruder test AS N = 9, WT N = 11. The stimulus mice were WT mice from WT breeding on the same C57BL/6 background for all experiments. Ages of stimulus mice are specified per each experiment.

All cages were kept on a 12 h light/dark cycle, and the behavioral experiments were consistently conducted during the light phase of the cycle. With the exception of the testing times, the mice had access to food and water ad libitum.

Experiments were approved by the University of Haifa Institutional Committee for animal experiments in accordance with National Institutes of Health guidelines.

### Three-chamber social test

#### Sociability test

An adult male mouse (‘subject mouse’; a C57BL/6 2–3 months of age), was placed in an empty arena for 30 min, a day before the test. On the test day, the subject mouse was placed in the center chamber with open partitions for a 10 min habituation period. Following the above, doors were blocked and an unfamiliar mouse (‘stranger 1’; a WT C57BL/6 4–8 week-old male mouse), was enclosed inside a wire cage in one of the side chambers (‘social chamber’). Identical wire cage was placed in the opposite side (‘object chamber’) and remained vacant. For illustration, see Fig. 1a.

The placement locations for stranger 1 and the empty wire cage were systematically alternated between the left and right chambers across subjects to prevent side-preference bias. Following placement of stranger 1, the doors were opened, and the subject was allowed to move freely and explore the entire arena for the duration of 10 min per test trial.

For comparison of the subjects' investigations of social versus object stimuli, we measured the time spent in each chamber and the time spent in sniffing each one of the wire cages. The time spent in each chamber was automatically scored by Ethovision XT10, and the sniffing time was scored by a blinded trained observer using two stopwatches. The social preference index (PI) was calculated as

$$\frac{{\left( {social\;wire\;cage\;sniffing\;time - object\;wire\;cage\;sniffing\;time} \right)}}{{\left( {social\;wire\;cage\;sniffing\;time + object\;wire\;cage\;sniffing\;time} \right)}}$$

The existence of a social preference is defined as a statistically significant difference from the chance level (PI = 0).

#### Social novelty test

Immediately after the sociability test, a new unfamiliar mouse (‘stranger 2’; a WT C57BL/6 4–8-week-old male mouse) was placed in the previously empty wire cage. The subject mouse was given an additional 10 min exploration period in order to measure its preference between the first (already-investigated stranger 1) ‘familiar mouse’ and the second, novel, ‘unfamiliar mouse’ (stranger 2). For illustration, see Fig. 1a.

For comparison of the subjects' investigations of unfamiliar versus familiar mouse, we measured the time spent in each chamber and the time spent in sniffing each one of the wire cages. The time spent in each chamber was automatically scored by Ethovision XT10 and the sniffing time was scored by a blinded trained observer using two stopwatches. The social novelty PI was calculated as

$$\frac{{\left( {unfamiliar\;wire\;cage\;sniffing\;time - familiar\;wire\;cage\;sniffing\;time} \right)}}{{\left( {unfamiliar\;wire\;cage\;sniffing\;time + familiar\;wire\;cage\;sniffing\;time} \right)}}$$

The existence of social novelty preference is defined as a statistically significant difference from the chance level (PI = 0). The non-littermates, stranger 1 and stranger 2, never had physical contact with the subject mice before. Several days prior to the commencement of the social preference testing, the stranger mice were habituated to the wire cages within the apparatus, in order to minimize their activity and prevent aggressiveness. The arena and wire cages were wiped down with water and 70% ethanol between each subject.

### Social habituation/dishabituation test

Each subject mouse (a C57BL/6 10-week-old male mouse) was placed in a testing cage containing a clean woodchip bedding, and moved to the testing room 30 min prior to the test. Subsequently, the subject mouse was exposed in a series of five successive short 1 min encounters with a conspecific stimulus (a WT C57BL/6 4–8-week-old male mouse). The encounters were separated by 10 min inter-trial intervals. In the first four trials (‘habituation phase’), the same stimulus mouse was repeatedly introduced to the subject mouse. Following the fourth exposure, a novel social stimulus was presented for the fifth and final trial (‘dishabituation phase’). In order to avoid aggression during the test, the stimulus mouse was placed inside a wire cage during the encounters (Fig. 3a). The cumulative duration that the subject mouse was engaged in social investigation with the stimulus mouse was manually scored using EthoX10 vision software by a blinded experimenter.

### Long-term social discrimination memory (SDM) test

An adult male mouse (a C57BL/6 2–3 months of age), was exposed to a novel mouse (considered as ‘familiar mouse’; a WT C57BL/6 4–8-week-old male mouse) placed in the wire cage located in the abovementioned arena without the dividers for 1 h (‘social familiarization phase’). The long-term SDM test was conducted 24 h following the social familiarization phase. The subject mouse was placed at the arena with two empty wire cages for a 15 min habituation trial. Next, the pre-exposed mouse (familiar), and another novel unfamiliar mouse (‘unfamiliar’; a WT C57BL/6 4–8-week-old male mouse) were placed randomly in the wire cages, and the subject was given an additional 10 min to explore the two mice (Fig. 4a).

For comparison of the subjects' investigations of unfamiliar versus familiar mice we measured the time spent in sniffing each one of the wire cages. The sniffing time was scored by a blinded trained observer using two stopwatches.

The long-term SDM PI to the unfamiliar was calculated as

$$\frac{{\left( {unfamiliar\;wire\;cage\;sniffing\;time - familiar\;wire\;cage\;sniffing\;time} \right)}}{{\left( {unfamiliar\;wire\;cage\;sniffing\;time + familiar\;wire\;cage\;sniffing\;time} \right)}}$$

The existence of a long-term SDM is defined as a statistically significant difference from the chance level (PI = 0).

### Resident-intruder aggression test

All subject mice (‘resident’; C57BL/6 male mice, 10–12 weeks old) were singly housed 10 days prior to testing. Stimulus mice (‘intruder’; a WT C57BL/6 male mice 7–8 weeks old, marked with dye on its back) were group housed and used for only a single encounter per day. At the day of the test, a weight-matched (< 5 g difference) intruder mouse was introduced into the resident's home cage for a 5 min encounter. Each mouse was given three tests with two days between each test. For each resident mouse, a new intruder mouse was used in each test (Fig. 5a).

All encounters were videotaped and subsequently manually scored by EthoX10 vision software by a blinded experimenter. An aggression attack was scored as whenever the resident animal approached the intruder and engaged in any agonistic behavior such as biting, chasing and clinch attack, according to previously described discrimination^37^. Only aggression that started by the resident mouse was scored. There were only two cases in which the aggression started by the intruder, and these two mice were excluded from the data. It is critical to note that these two mice were AS mice, and this did not happen with WT mice. The latency to the first attack, cumulative attack duration, and frequency of the attacks initiated by the resident were scored. Subjects who did not exhibit attacks during the 5 min trial had 300 s recorded as their latency score. In addition, same parameters were recorded for clinch attack separately and to social exploration as well.

### Statistical analysis

Statistical analyses were performed using GraphPad Prism 7. Data were analyzed by parametric unpaired t-tests and two-way repeated measures ANOVA followed by Bonferroni's multiple comparisons tests. For all comparisons between groups or conditions, significance was set at *p* < 0.05 using t-tests or ANOVAs wherever necessary. We also performed one-sample t-tests comparisons to chance level, which to depict the significance of a certain preference. In addition, correlations were performed using Pearson correlation in cases of normal distribution, and non-parametric Spearman correlation when distributions did not pass normality tests. Results are displayed as mean ± SEM in all graphs.

## Supplementary Information

Supplementary Information.

Supplementary Information.

Supplementary Information.
