# Supplementary figures and images for: Aberrant aggressive behavior in a mouse model of Angelman syndrome
Source: Sci Rep. 2021 Jan 8;11:47. doi: 10.1038/s41598-020-79984-7 (PMC7794213; doi:10.1038/s41598-020-79984-7)

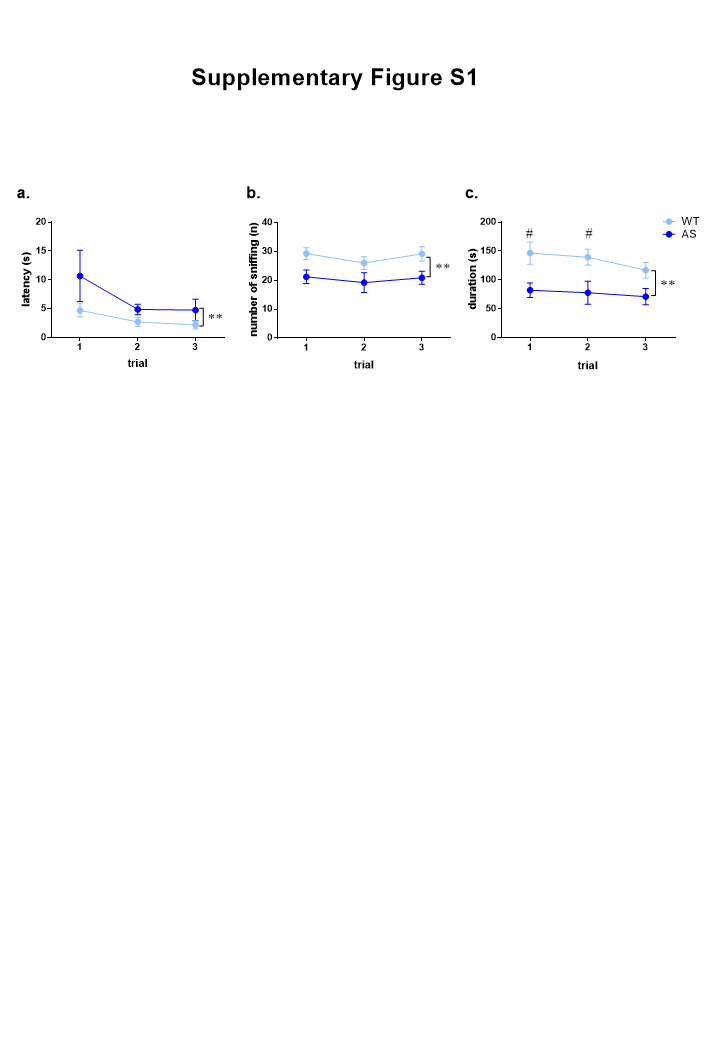

Supplement: Supplementary file 1 — Supplementary Information. [file 41598_2020_79984_MOESM1_ESM.tif]
