# Supplementary material for: Aberrant aggressive behavior in a mouse model of Angelman syndrome
Source: Sci Rep. 2021 Jan 8;11:47. doi: 10.1038/s41598-020-79984-7 (PMC7794213; doi:10.1038/s41598-020-79984-7)
Supplement: Supplementary file 2 — Supplementary Information. [file 41598_2020_79984_MOESM2_ESM.pdf]

Supplementary Table 1: Correlations between weight and distinct offensive behavior parameters in the resident-intruder test.

a.

	weight VS. aggression frequency- 1st trial		weight VS. aggression duration- 1st trial		weight VS. aggression latency- 1st trial		weight VS. aggression frequency- 2nd trial		weight VS. aggression duration- 2nd trial		weight VS. aggression latency- 2nd trial		weight VS. aggression frequency- 3rd trial		weight VS. aggression duration- 3rd trial		weight VS. aggression latency- 3rd trial	
	r	Sig.	r	Sig.	r	Sig.	r	Sig.	r	Sig.	r	Sig.	r	Sig.	r	Sig.	r	Sig.
WT	0.48	0.13	0.1	0.77	-0.38	0.25	0.08	0.81	0.04	0.9	0.03	0.93	0.02#	0.97	0.14	0.68	-0.1	0.77
AS	0.15	0.69	0.32	0.4	-0.21 #	0.58	0.44	0.23	0.59	0.09	-0.1	0.79	0.12 #	0.76	0.52	0.16	-0.46	0.21

b.

	weight VS. clinch frequency- 1st trial		weight VS. clinch duration- 1st trial		weight VS. clinch latency- 1st trial		weight VS. clinch frequency- 2nd trial		weight VS. clinch duration- 2nd trial		weight VS. clinch latency- 2nd trial		weight VS. clinch frequency- 3rd trial		weight VS. clinch duration- 3rd trial		weight VS. clinch latency- 3rd trial	
	r	Sig.	r	Sig.	r	Sig.	r	Sig.	r	Sig.	r	Sig.	r	Sig.	r	Sig.	r	Sig.
WT	x	x	x	x	x	x	-0.43	0.18	-0.28 #	0.41	0.16#	0.64	-0.26 #	0.82	-0.26 #	0.82	0.26#	0.82
AS	-0.24	0.53	0.17	0.66	0.13	0.74	-0.13	0.74	0.17	0.67	0.14	0.72	-0.11	0.78	0.24 #	0.54	-0.3	0.43

X = no correlation is possible because all the values were similar.

# = Nonparametric Spearman correlation due to non-normal distribution of the data.
