# Supplementary material for: Aberrant aggressive behavior in a mouse model of Angelman syndrome
Source: Sci Rep. 2021 Jan 8;11:47. doi: 10.1038/s41598-020-79984-7 (PMC7794213; doi:10.1038/s41598-020-79984-7)
Supplement: Supplementary file 3 — Supplementary Information. [file 41598_2020_79984_MOESM3_ESM.docx]

**Supplementary Figure 1: AS male mice exhibit reduced affiliative social exploration during the resident-intruder test.**

(a-c) AS mice engage less in sniffing the intruder mouse compared to their WT littermates. (a) The latency to the first sniffing (b) Number of sniffing events during 5 min. (c) Total accumulative duration of sniffing the intruder mouse by the resident mouse. Line graphs represent mean ± SEM. N=11 mice, N=9 mice for WT and AS, respectively. ^#^p<0.05 represents a genotype difference for each trial; **p<0.01 represents an accumulative genotype effect.
